# Environmental Influences on Mate Preferences as Assessed by a Scenario Manipulation Experiment

**DOI:** 10.1371/journal.pone.0074282

**Published:** 2013-09-12

**Authors:** Daniele Marzoli, Francesco Moretto, Aura Monti, Ornella Tocci, S. Craig Roberts, Luca Tommasi

**Affiliations:** 1 Dipartimento di Scienze Psicologiche, Umanistiche e del Territorio, University of Chieti, Chieti, Italia; 2 School of Natural Sciences, University of Stirling, Stirling, Scotland, United Kingdom; University of Goettingen, Germany

## Abstract

Many evolutionary psychology studies have addressed the topic of mate preferences, focusing particularly on gender and cultural differences. However, the extent to which situational and environmental variables might affect mate preferences has been comparatively neglected. We tested 288 participants in order to investigate the perceived relative importance of six traits of an ideal partner (wealth, dominance, intelligence, height, kindness, attractiveness) under four different hypothetical scenarios (status quo/nowadays, violence/post-nuclear, poverty/resource exhaustion, prosperity/global well-being). An equal number of participants (36 women, 36 men) was allotted to each scenario; each was asked to allocate 120 points across the six traits according to their perceived value. Overall, intelligence was the trait to which participants assigned most importance, followed by kindness and attractiveness, and then by wealth, dominance and height. Men appraised attractiveness as more valuable than women. Scenario strongly influenced the relative importance attributed to traits, the main finding being that wealth and dominance were more valued in the poverty and post-nuclear scenarios, respectively, compared to the other scenarios. Scenario manipulation generally had similar effects in both sexes, but women appeared particularly prone to trade off other traits for dominance in the violence scenario, and men particularly prone to trade off other traits for wealth in the poverty scenario. Our results are in line with other correlational studies of situational variables and mate preferences, and represent strong evidence of a causal relationship of environmental factors on specific mate preferences, corroborating the notion of an evolved plasticity to current ecological conditions. A control experiment seems to suggest that our scenarios can be considered as realistic descriptions of the intended ecological conditions.

## Introduction

Many evolutionary psychology studies have addressed the topic of mate preferences focusing mainly on gender and cultural factors (e.g., [1]–[3]). Across different cultures, women’s and men’s mate preferences show some similarities, both sexes preferring partners who are intelligent, kind, understanding and healthy, and that share their values [4]. However, women and men faced rather different selection pressures during human evolution, and consequently they also show noteworthy dissimilarities [5].

The possession of economic resources and related attributes (such as social status, ambition and industry) are characteristics of a prospective mate that are almost universally appreciated more by women than by men. For the same reason, women tend to prefer mates who are older than they are. Overall, this suggests that women’s preferences have been shaped by selection to target men who are more able to provide resources [5]. On the contrary, men from different cultures are particularly attracted by women’s youth and physical attractiveness, likely because these qualities have been linked with women’s fertility during human evolution [5].

Although mate preferences have been shaped by selection, they are not fixed, but suitably flexible, depending on a number of different factors. For example, it has been found that women and men with high mate value (that is, attractiveness as a partner) show a strong preference for partners with high mate value, whereas people with low mate value are less choosy, and this is found in both laboratory (e.g., [6]–[10]) and naturalistic studies (e.g., [11]–[14]). Mate preferences can also be influenced by the type of relationship sought (e.g., [6], [9], [10], [12], [15], [16]). Moreover, mate preferences can be modulated by hormone levels in selectors (e.g., [17]–[22]), as well as by hormone markers in potential selectees (e.g., [17], [21], [23]–[25]).

### Environmental Factors in Mate Preferences

Mate preferences can also be strongly affected by situational and environmental variables. For example, compared to individuals living in areas with low pathogen prevalence, those living in areas with high pathogen prevalence place greater importance on a mate’s physical attractiveness, a trait associated with pathogen resistance [26]. Furthermore, a cross-cultural study showed that women’s preference for men’s facial masculinity – a trait linked to good health – is negatively correlated with average national health [27] (see also [28], [29]). In line with these correlational studies, recent experimental evidence shows that women’s mate preferences shift towards good-genes traits or traits indicating high paternal investment when participants are primed with pathogen prevalence and resource scarcity, respectively [30]. Similarly, exposure to visual cues of environmental pathogens increases preferences for mates exhibiting health-related traits, so that women prefer more masculine and symmetrical male faces and men prefer more feminine and symmetrical female faces [31]. Finally, a recent study [32] has shown that exposure to visual environmental cues of direct male-male competition, violence and wealth also increases women’s preferences for masculine male faces.

Environmental factors, and geographical location in particular, can also influence mate preferences for traits other than physical appearance. For example, women who live in cities in which the cost of living is high demand more resources and fewer emotional qualities in a prospective mate within their personal advertisements [33]. In keeping with such findings, people from less socioeconomically developed countries rate the possession of characteristics linked to resource acquisition as more important in a long-term mate, and consider mutual attraction/love as less important, compared to individuals from more developed countries [34]. Importantly, Eagly and Wood [35] observed that, across cultures, women’s access to resources and power inversely predicts the extent to which women emphasize (compared to men) a potential spouse's earning capacity. Similarly, Zentner and Mitura [36] found that gender differences in adaptive mate preferences decline proportionally to increases in nations’ gender equality (however, see [37] for a methodological criticism).

On the whole, the reviewed studies suggest – as also argued by Little et al. [31], [32] – that people (or at least women) prefer partners exhibiting resource-related and health-related traits under environmental conditions of low and high resources, respectively (see also [38]), whereas they prefer partners exhibiting health-related and resource-related traits under environmental conditions of high and low pathogen prevalence, respectively (see [39]).

### The Budget Allocation Method

The relative importance attached to different characteristics of an ideal mate also depends on the resources available to attain those characteristics. Li, Bailey, Kenrick, and Linsenmeier [40] devised an ingenious method to investigate which characteristics of a potential partner are judged by women and men as necessities, and which as luxuries. The researchers gave their participants varying budgets (low, medium and high) of “mate dollars” to be spent in “designing” their ideal long-term mates with regard to a set of discrete qualities. They observed that, when given a low budget, women spent large proportions of it on economic status and intelligence, and men on physical attractiveness and intelligence. However, when the budget increased, women and men spent larger proportions on other characteristics such us kindness and creativity. In summary, economic status and intelligence appear to be prioritized by women, while physical attractiveness and intelligence are prioritized by men. Kindness is essential to both sexes, whereas creativity and other qualities can be considered as luxuries (see [7] for similar results).

Employing a methodological approach similar to that of Li et al. [40], Waynforth [41] found that women – but not men – trade off physical attractiveness for resources, and suggested that this could partly explain why attractiveness plays a lesser role in determining men’s mate value. Waynforth argues that the link between resource-based parental investment and offspring fitness might decrease when levels of investment increase, and that once a certain resource acquisition ability or accrual point is reached, male resources would have less impact on female fitness. As a consequence, women should begin to rate potential mates primarily according to their physical attractiveness.

### The Present Study

It is noteworthy that most previous research concerning the effects of environmental variables on mate preferences have been correlational in nature [26]–[28], [33]–[36], with environmental variables not being manipulated experimentally (however, see [30]–[32] for studies employing priming paradigms). As also stressed by Lee and Zietsch [30], correlational studies cannot demonstrate a direct causal relationship between specific mate preferences and environmental factors, nor can they discriminate between whether environmental factors change the genetic component of mate preferences by means of selection pressures over time, or whether changes in mate preferences occur by virtue of an evolved plasticity to environmental factors.

Some researchers have addressed the effects of manipulating situational variables by investigating mate preferences in different hypothetical scenarios, but their manipulation either merely addressed the effects of different locations (more or less supportive of sex-specific reproductive goals) on women’s and men’s mate preferences for a one-night stand [42] or included participants’ personal characteristics such as educational level and occupational status [43],[44], thus possibly affecting also their own perceived mate value, a trait known to influence mate preferences (e.g., [6]–[14]; in particular, see [7], in which a budget allocation paradigm was used). In view of this, the present study aimed to cast more light on the effects of environmental factors by using different virtual scenarios, but without intentionally manipulating the perception of participants’ own mate value.

We employed a method similar to that of Li et al. [40], which we believe to be particularly suitable for detecting differences in the importance assigned to various traits in different conditions: while simply asking participants to evaluate the importance of different traits can lead them to maximally rate a number of traits in each scenario, a method necessarily involving a trade-off in importance ratings is more likely to yield differences in the relative importance of different traits in different scenarios.

In a first study (Experiment 1), we combined the manipulation of virtual scenarios with the budget allocation method in order to investigate the relative importance that women and men assign to six traits of an ideal partner (wealth, dominance, intelligence, height, kindness and attractiveness), under four different scenarios (nowadays, post-nuclear, resource exhaustion and global well-being).

Based on previous studies, we hypothesized that:

1. compared to the opposite sex, female participants should show a stronger preference for wealthy partners, and male participants for attractive partners, whereas no significant sex differences should emerge with respect to intelligence and kindness (see [1], [40]);
2. participants should show a stronger preference for partners possessing wealth (namely, resources to be invested in their mate and/or offspring) in scenarios describing environments with fewer resources than in those describing environments with more resources (see [33]–[35]);
3. participants (at least women) should show a stronger preference for attractive partners in high resource compared to low resource scenarios, because people are more likely to be able to obtain, by themselves, the resources they need in a rich environment than in a poor environment (see [41]; [45] for a review);
4. female participants should show a stronger preference for tall and dominant partners in the most socially violent scenario than in the other scenarios, in line with the bodyguard hypothesis [46], according to which women’s preference for physically and socially dominant mates would represent an evolutionary adaptation to the need for protection from aggressive men (see also [47], which observed that married women incur less risk of both lethal and nonlethal sexual aggression than unmarried women, and [48], which found that women’s fear of crime positively correlates with their preferences for aggressive and formidable mates).

We did not have specific hypotheses concerning the effects of scenario manipulation on intelligence and kindness.

A second study (Experiment 2) was carried out in order to test whether our scenarios can be considered as realistic descriptions of the intended ecological conditions.

## Experiment 1

### Method

We tested 288 subjects (144 females and 144 males). The sample consisted of 248 participants who were students and/or apprentices, 32 who were in employment, four who were unemployed, and four who did not indicate their occupation. Participants were all Caucasian, were recruited on a voluntary basis, and were tested on the university campus (187 subjects), at cinemas (14 subjects), bars or pubs (14 subjects), home (nine subjects), swimming pools (four subjects) and in other unspecified places (60 subjects). Participants were required to give only oral consent because neither invasive nor risky procedures were involved and because the data were analyzed anonymously; their written responses were used to document their consent. The study was carried out in accordance with the principles of the Declaration of Helsinki and was approved (including the oral consent process) by the local ethical committee (Comitato Etico d’Ateneo, Università “G. d’Annunzio” – Chieti).

Potential participants were approached by a female or male experimenter and asked to take part in a short and anonymous study (participation was conditional on not being currently in a long-term relationship). If the subject gave her/his consent, the experimenter provided her/him with a paper sheet with a written request to (a) imagine suddenly finding her/himself in one of four scenarios and (b) attribute a number of points (out of a budget of 120) to each of six traits, according to their relevance, in the context of searching for a potential partner in that scenario (the only constraint was that they should spend the whole budget). After completing the task, participants completed a brief questionnaire to obtain basic demographic information (sex, age and sexual orientation).

An equal number of participants (36 females and 36 males) was allotted to each of the following scenarios: status quo/nowadays scenario, violence/post-nuclear scenario, poverty/resource exhaustion scenario and prosperity/global well-being scenario (the narratives describing the four scenarios are reported in Appendix S1 of the Supporting Information). Because past research indicates that the experimenter’s sex can affect participants’ reported sex-related attitudes and behaviors (e.g., [49]–[52]), half of the participants in each experimental condition were tested by a female experimenter and the other half by a male experimenter. The order (from top to bottom) of the six traits (wealth, dominance, intelligence, height, kindness and attractiveness; in Italian, respectively, ‘ricchezza’, ‘dominanza’, ‘intelligenza’, ‘altezza’, ‘gentilezza’ and ‘bellezza’) in the response sheet was fully balanced across experimental conditions.

We performed a mixed model ANOVA employing Participant’s Sex, Experimenter’s Sex and Scenario as between-subjects factors, and Trait as the within-subjects factor. When a significant effect was found, a Bonferroni-Holm correction [53] for multiple comparisons was applied to each set of post-hoc comparisons. Unlike the Bonferroni correction, the Bonferroni-Holm procedure (Holm, 1979) allows correction of the alpha value – step-by-step – every time a significant difference is found: this method starts with the standard Bonferroni adjustment for the first test, but increases the significance level for the following ones by changing the alpha value according to the number of remaining comparisons.

### Results

Seventeen participants (11 women and six men) were excluded from data analysis because they indicated they were not heterosexual. In addition, as different budget amounts can influence spending patterns for the traits desired in a mate [40], 13 participants (six women and seven men) were also excluded because they spent either more or less of the available budget. Finally, we excluded 33 women and 26 men who scored, on any trait, more than 2 standard deviations above or below the mean according to their “Sex x Scenario” group (i.e., participants who allocated extreme numbers of points to any trait in any experimental condition), because we felt this might indicate less than full engagement with the task. Thus, our final sample consisted of 94 women (prosperity: *N* = 23; status quo: *N* = 23; violence: *N* = 22; poverty: *N* = 26) aged 18–30 years (*M* = 21.21±2.64 *SD*) and 105 men (prosperity: *N* = 27; status quo: *N* = 26; violence: *N* = 26; poverty: *N* = 26) aged 18–38 (*M* = 22.35±3.72 *SD*).

The effects of the interactions including Experimenter’s Sex were not significant.

The effect of Trait was significant (*F*_5,915_ = 209.90; *p*<0.001). A series of post-hoc comparisons (*N* = 15) showed that intelligence was assigned more points than kindness, attractiveness, wealth, dominance and height; moreover, kindness and attractiveness were assigned more points than wealth, dominance and height; finally, wealth was assigned more points than height (three comparisons were not significant).

**Figure 1:**
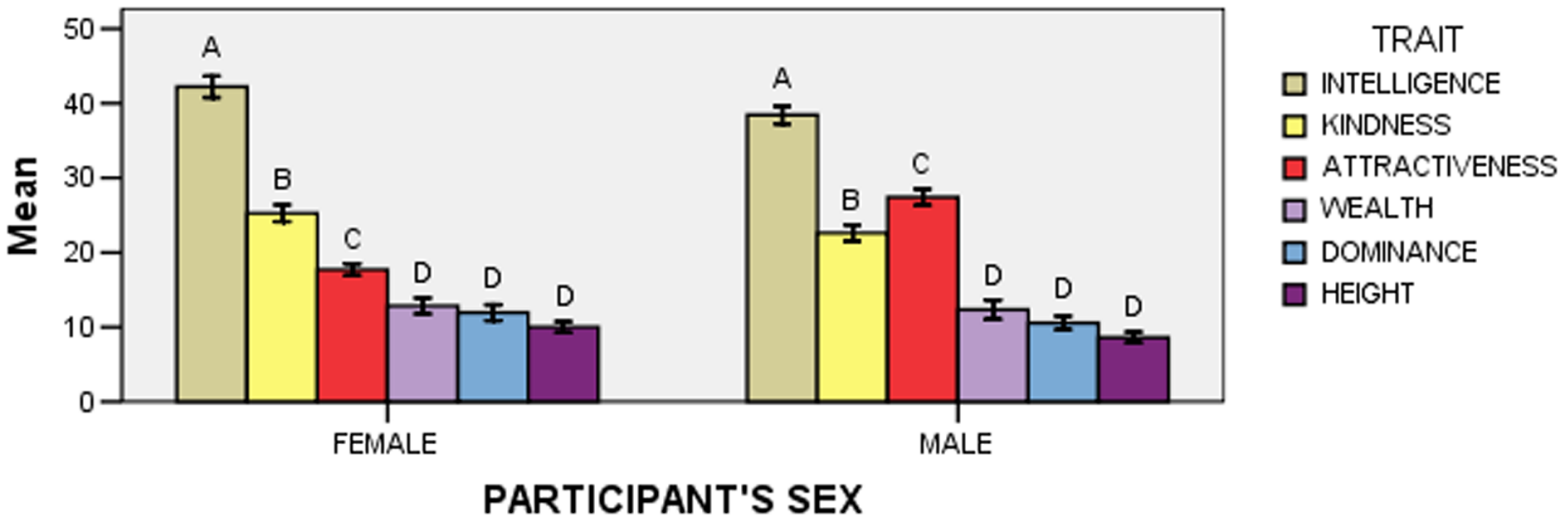
Mean points (± *SE*) allocated to each trait according to participants’ sex in Experiment 1. Within each “Participant’s Sex” group, means with different letters are significantly different from one another, as determined by Bonferroni-Holm post-hoc comparisons.

The effect of the Trait x Participant’s Sex interaction was significant (*F*_5,915_ = 9.57; *p*<0.001; Figure 1). A first series of post-hoc comparisons (*N* = 6) examined whether points assigned to any trait differed between female and male participants, and showed that attractiveness was assigned more points by men than by women (five comparisons were not significant).

A second series of post-hoc comparisons (*N* = 30) examined whether the pattern of point allocation differed according to participants’ sex, and showed that women assigned more points to kindness than to attractiveness, whereas men assigned more points to attractiveness than to kindness (see Figure 1 for all significant differences; six comparisons were not significant).

**Figure 2:**
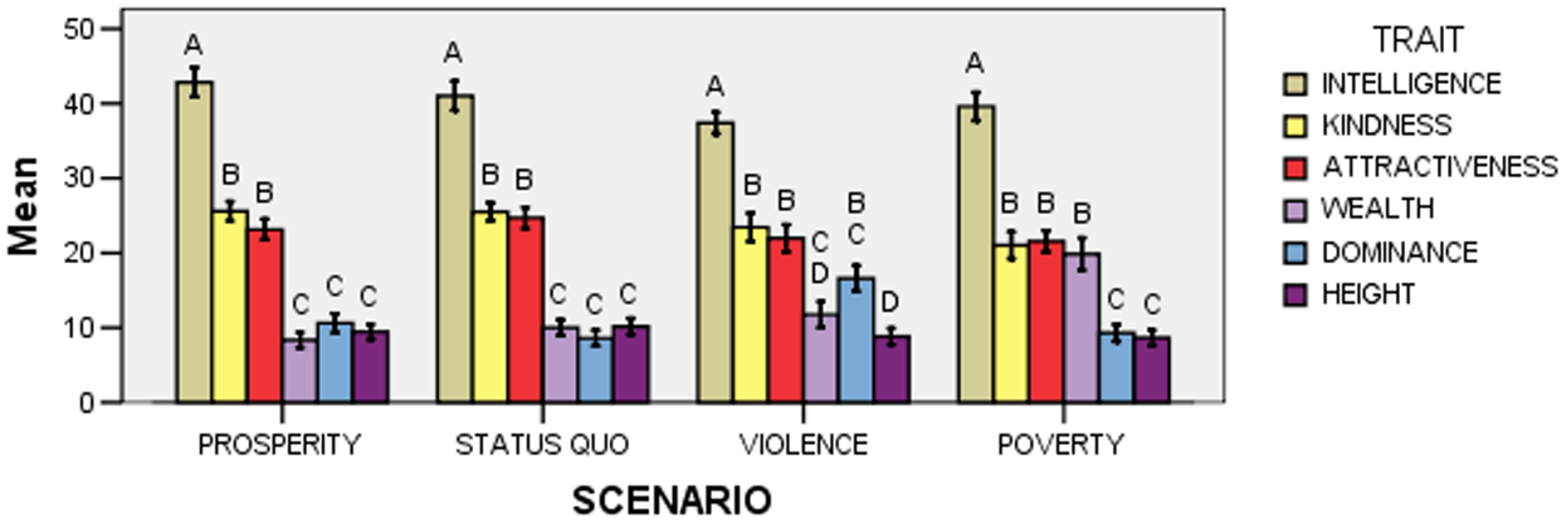
Mean points (± *SE*) allocated to each trait according to scenario in Experiment 1. Within each “Scenario” group, means with different letters are significantly different from one another, as determined by Bonferroni-Holm post-hoc comparisons.

The effect of the Trait x Scenario interaction was significant (*F*_15,915_ = 4.40; *p*<0.001; Figure 2). A first series of post-hoc comparisons (*N* = 36) examined whether points assigned to any trait differed between scenarios, and showed that wealth was assigned more points in the poverty scenario than in all other scenarios, whereas dominance was assigned more points in the violence scenario than in the status quo and poverty scenarios (31 comparisons were not significant; however, an almost significant difference was observed between points assigned to dominance in the violence and prosperity scenarios, with *p* = 0.00162>0.05/31).

A second series of post-hoc comparisons (*N* = 60) examined whether the pattern of point allocation differed according to scenario, and showed that kindness and attractiveness were assigned more points than wealth in all but the poverty scenario, and more points than dominance in all but the violence scenario; moreover, in the violence scenario, dominance was assigned more points than height; finally, in the poverty scenario, wealth was assigned more points than dominance and height (see Figure 2 for all significant differences; 17 comparisons were not significant).

**Figure 3:**
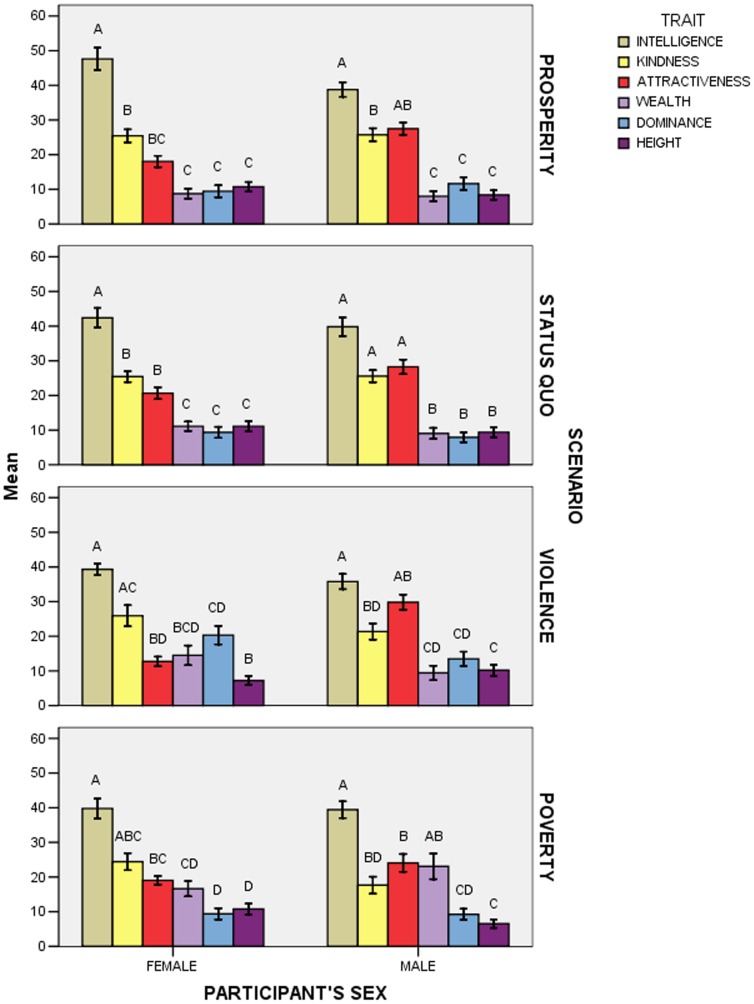
Mean points (± *SE*) allocated to each trait according to participants’ sex and scenario in Experiment 1. Within each “Participant’s Sex x Scenario” group, means with different letters are significantly different from one another, as determined by Bonferroni-Holm post-hoc comparisons.

The effect of the Trait x Scenario x Participant’s Sex interaction was significant (*F*_15,915_ = 2.05; *p* = 0.010; Figure 3). A first series of post-hoc comparisons (*N* = 72) examined whether points assigned to any trait differed between scenarios in female and male participants, and showed that women assigned more points to dominance in the violence scenario than in all other scenarios, whereas men assigned more points to wealth in the poverty scenario than in all other scenarios; moreover, women assigned fewer points to attractiveness in the violence scenario than in the status quo scenario (65 comparisons were not significant).

A second series of post-hoc comparisons (*N* = 24) examined whether points assigned to any trait differed between female and male participants in the different scenarios, and showed that in the prosperity and violence scenarios attractiveness was assigned more points by men than by women (22 comparisons were not significant).

A third series of post-hoc comparisons (*N* = 120) examined whether the pattern of point allocation differed according to scenario and participants’ sex, and showed that women assigned more points to kindness than to dominance in all but the violence scenario, whereas men assigned more points to intelligence and attractiveness than to wealth in all but the poverty scenario; moreover, in the violence scenario, women assigned more points to dominance than to height, and, in the poverty scenario, men assigned more points to wealth than to height (see Figure 3 for all significant differences; 51 comparisons were not significant).

### Discussion

Our data indicate that participants assigned most importance to intelligence, followed by kindness and attractiveness, and then by wealth, dominance, and height. A very similar pattern was observed in both women and men, with a few differences: (a) attractiveness was more important for men than for women; (b) kindness was more important than attractiveness for women, whereas attractiveness was more important than kindness for men. Overall, these findings are in line with those from other studies employing the budget allocation method (e.g., [7], [40]), which report intelligence, attractiveness and kindness as being among the most preferred traits in a potential mate (see also [4], [5]). The fact that the importance of attractiveness relative to kindness was higher in men (and vice versa in women) is consistent with previous research [2]. However, we failed to observe the often-reported sex difference according to which women more than men desire partners with economic resources, perhaps due to the scenario manipulation masking some sex differences specific to the current ecological conditions (see Experiment 2 for a more detailed discussion).

Albeit not predicted, the fact that our participants, of both sexes, assigned the greatest importance to intelligence is consistent with the study of Buss et al. [2] showing that, among a set of 13 characteristics, both female and male Italian subjects ranked intelligence as the most desirable trait in a mate (see also [3] for similar findings in a sample of participants from 53 countries across the world). This result could perhaps be ascribed to the ubiquitous importance of intelligence in problem-solving, regardless of environmental context.

Although the patterns of preferences were quite similar across scenarios, roughly resembling that of the total sample, the manipulation of environmental factors did significantly influence participants’ responses. Perhaps not surprisingly, the most consistent changes induced by the varying scenarios concerned wealth and dominance. The greater importance attributed to wealth in the poverty scenario is in agreement with reports that people from less socioeconomically developed countries attach greater value to characteristics linked to resource acquisition [34] (see also [33], [35]). However, one could ask why participants presented with the description of the other environment with scarce resources (i.e., the violence scenario) did not similarly exhibit an increased preference for wealthy partners compared with those participants presented with the descriptions of more prosperous environments. A possible account is that, in a situation where human relationships are not ruled by law but by brute force, the possession of resources by one’s own mate is not the primary factor influencing survival and fitness. In contrast, in such a situation, one could take much more advantage of a dominant partner, and this view is supported by our finding that participants attributed greater importance to dominance in the violence scenario compared to all other conditions, in line with the bodyguard hypothesis [46], [47]. The fact that dominance was largely disregarded in all non-violent environments strongly corroborates the proposal of Snyder et al. [48] that, because interpersonal aggression towards same-sex and opposite-sex individuals are highly correlated [54] and the use of aggression for personal gain outside of the home predicts partner abuse [55], [56], dominant – and thus probably aggressive – mates are preferred only when they are really needed, that is under conditions in which it can be expected that the costs of partnering with aggressive individuals are outweighed by the benefits that such individuals provide. This interpretation might account not only for the positive correlation found by Snyder et al. [48] between women’s fear of crime and their preferences for aggressive and formidable mates, but also for that found by Phelan, Sanchez and Broccoli [57] between fear of crime and the endorsement of benevolent sexism, which carries both costs and benefits for women because it perpetuates the status quo of male dominance by enhancing the belief that women need to rely on men for protection (e.g., [58], [59]). On the other hand, one could wonder why the preference for dominance observed in the violent scenario was not coupled with a parallel preference for height, because taller individuals are perceived to be more dominant [60] and possessing higher status [61], [62]. We postulate that this missing association might be due to height being a means by which to acquire dominance, rather than an end in itself. In other words, height could be desired as a cue – but not a guarantee – to dominance, and thus when one can choose between such a cue and dominance itself, the latter is preferred. The scenario manipulation did not affect the importance attributed to intelligence, kindness, attractiveness (maybe at odds with [41]; but see [33], [34] for similar results) and height. Contrary to our results, some previous research has found that individuals place greater importance on intelligence in environments with fewer resources; however, in those investigations, intelligence was either included in a broader factor (resource-holding potential; [33]) or coupled with a second quality (education; [34]).

In summary, according to our data, intelligence, kindness and attractiveness might be considered as necessities, holding the first three positions in all scenarios, without variation in relative importance across conditions. However, the relevance of wealth and dominance was critically affected by the scenario manipulation, with these being important only in the poverty and violence scenarios, respectively. In comparison, height was always least prioritized, regardless of scenario. Mate preferences did not differ between the two scenarios describing environments with more resources (prosperity and status quo), suggesting that the relative descriptions of environmental conditions were perceived as essentially overlapping.

Our data also suggest that the effects of environmental variables are quite similar for both sexes. This seems to be congruent with the findings of Stone et al. [34], who observed few sex-differentiated shifts in mate preferences according to socioeconomic environment. Similarly, Buss et al. [2] found that the effects of culture are stronger than those of sex in shaping mate preferences, which suggests more similarity between men and women from the same culture than between same-sex individuals from different cultures. Nonetheless, participants’ sex significantly interacted with scenario in determining some specific patterns of trait preferences. In particular, in the violence scenario, women attributed more importance to dominance compared to all other conditions, whereas in the poverty scenario, men attributed more importance to wealth compared to all other conditions. This suggests that women would be particularly prone to trade off other traits for dominance in violent environments, whereas males would be particularly prone to trade off other traits for wealth in poor environments.

## Experiment 2

A possible criticism of our experiment is that our scenarios might turn out not to be plausible descriptions of the intended ecological conditions, so we carried out a control experiment with the aim of comparing participants’ responses in the status quo scenario (intended to represent a future world as similar as possible to the current one) with preferences for an ideal partner of individuals simply required to answer one of two questions (phrased with slightly different words).

### Method

We tested 144 subjects (72 females and 72 males). Participants were required to give only oral consent because neither invasive nor risky procedures were involved and because the data were analyzed anonymously; their written responses were used to document their consent. The study was carried out in accordance with the principles of the Declaration of Helsinki and was approved (including the oral consent process) by the local ethical committee (Comitato Etico d’Ateneo, Università “G. d’Annunzio” – Chieti).

Potential participants were approached by a female or male experimenter and asked to take part in a short and anonymous study (participation was conditional on not being currently in a long-term relationship). If the subject gave her/his consent, the experimenter provided her/him with a paper sheet with a written request to attribute a number of points (out of a budget of 120) to each of six traits, according to their relevance, for a potential partner (the only constraint was that they should spend the whole budget). After completing the task, participants completed a brief questionnaire to obtain basic demographic information (sex, age and sexual orientation).

An equal number of participants (36 females and 36 males) was allotted to each of the following questions: (1) “If you were given the opportunity to choose, which characteristics would you prefer in a potential partner?” and (2) “If you now had the opportunity to choose, which characteristics would you prefer in a potential partner?” Because past research indicates that the experimenter’s sex can affect participants’ reported sex-related attitudes and behaviors (e.g., [49]–[52]), half of the participants in each experimental condition were tested by a female experimenter and the other half by a male experimenter. The order (from top to bottom) of the six traits (wealth, dominance, intelligence, height, kindness and attractiveness) in the response sheet was fully balanced across experimental conditions.

Data analysis included 216 participants (the 144 participants from the control experiment and the 72 participants from the status quo scenario of the main experiment), with the factor Question Type (questions 1 and 2 from the control experiment and status quo scenario from Experiment 1) replacing the factor Scenario. The sample consisted of 183 participants who were students and/or apprentices, 29 who were in employment, two who were unemployed, and two who did not indicate their occupation. Participants were all Caucasian, were recruited on a voluntary basis, and were tested on the university campus (133 subjects), at cinemas (11 subjects), bars or pubs (10 subjects), home (10 subjects) and in other unspecified places (52 subjects).We performed a mixed model ANOVA employing Participant’s Sex, Experimenter’s Sex and Question Type as between-subjects factors, and Trait as the within-subjects factor. When a significant effect was found, a Bonferroni-Holm correction for multiple comparisons was applied to each set of post-hoc comparisons.

### Results

Nine participants (five women and four men) were excluded from data analysis because they indicated they were not heterosexual. In addition, as different budget amounts can influence spending patterns for the traits desired in a mate [40], six participants (one woman and five men) were also excluded because they spent either more or less of the available budget. Finally, we excluded 28 women and 22 men who scored, on any trait, more than 2 standard deviations above or below the mean according to their “Sex x Question Type” group (i.e., participants who allocated extreme numbers of points to any trait in any experimental condition), because we felt this might indicate less than full engagement with the task. Thus, our final sample consisted of 74 women (question 1: *N* = 28; question 2: *N* = 23; status quo: *N* = 23) aged 18–29 (*M* = 21.57±2.73 *SD*) and 77 men (question 1: *N* = 27; question 2: *N* = 24; status quo: *N* = 26) aged 18–40 (*M* = 22.56±4.33 *SD*).

The effects of the interactions including Question Type were not significant.

The effect of Trait was significant (*F*_5,695_ = 243.21; *p*<0.001). A series of post-hoc comparisons (*N* = 15) showed that intelligence was assigned more points than attractiveness, kindness, height, dominance and wealth; moreover, attractiveness and kindness were assigned more points than height, dominance and wealth (four comparisons were not significant).

**Figure 4:**
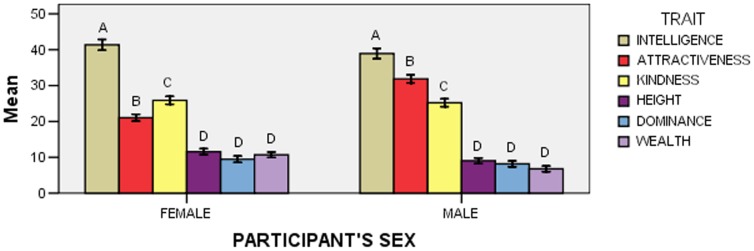
Mean points (± *SE*) allocated to each trait according to participants’ sex in Experiment 2. Within each “Participant’s Sex” group, means with different letters are significantly different from one another, as determined by Bonferroni-Holm post-hoc comparisons.

The effect of the Trait x Participant’s Sex interaction was significant (*F*_5,695_ = 11.39; *p*<0.001; Figure 4). A first series of post-hoc comparisons (*N* = 6) examined whether points assigned to any trait differed between female and male participants, and showed that wealth was assigned more points by women than by men, whereas attractiveness was assigned more points by men than by women (four comparisons were not significant).

A second series of post-hoc comparisons (*N* = 30) examined whether the pattern of point allocation differed according to participants’ sex, and showed that women assigned more points to kindness than to attractiveness, whereas men assigned more points to attractiveness than to kindness (see Figure 4 for all significant differences; six comparisons were not significant).

**Figure 5:**
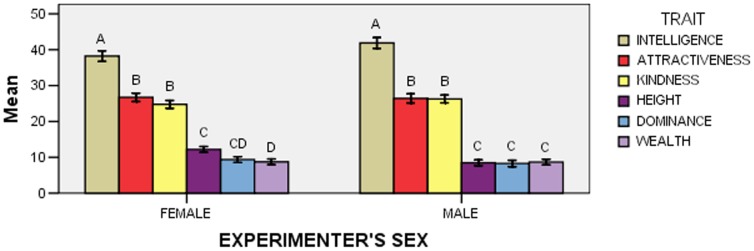
Mean points (± *SE*) allocated to each trait according to experimenter’s sex in Experiment 2. Within each “Experimenter’s Sex” group, means with different letters are significantly different from one another, as determined by Bonferroni-Holm post-hoc comparisons.

The effect of the Trait x Experimenter’s Sex interaction was significant (*F*_5,695_ = 2.47; *p* = 0.031; Figure 5). A first series of post-hoc comparisons (*N* = 6) examined whether points assigned to any trait differed between participants tested by either a female or male experimenter, and showed that height was assigned more points by participants tested by a female experimenter than by those tested by a male experimenter (five comparisons were not significant).

A second series of post-hoc comparisons (*N* = 30) examined whether the pattern of point allocation differed according to experimenter’s sex, and showed that participants tested by a female experimenter assigned more points to height than to wealth (see Figure 5 for all significant differences; seven comparisons were not significant).

### Discussion

Our data show that the particular way of phrasing the question did not affect participants’ preferences for an ideal partner when ecological conditions were not specifically manipulated relative to the current ones. Overall, the pattern of preferences was almost identical to that observed in the main experiment, participants assigning most importance to intelligence, followed by attractiveness and kindness, and then by height, dominance and wealth. A very similar pattern was also observed in both women and men, with a few differences: (a) wealth was more important for women than men, while attractiveness was more important for men than women; (b) kindness was more important than attractiveness for women, while attractiveness was more important than kindness for men. As with the results from the main experiment, these findings are in line with those from other studies employing the budget allocation method (e.g., [7], [40]), according to which intelligence, attractiveness and kindness are among the most preferred traits in a potential mate (see also [4], [5]). As in the main experiment, the importance of attractiveness relative to kindness was higher and lower, respectively, in men and women, consistent with previous research [2]. However, in contrast to the main experiment, we also observed that women showed a stronger preference than men for wealth, as often reported in the past [2], [5]. A possible account for such a discrepancy might be that our scenario manipulation could have masked the least robust sex differences, an explanation which is consistent with studies indicating that ecological factors such as culture and socioeconomic environment seem to play a greater role than sex in shaping mate preferences [2], [34]. In the same vein, it is also worth noting that past research has usually investigated mate preferences in environments quite similar to the current one and, presumably, to that described in the status quo scenario. Finally, participants tested by a female experimenter attributed more importance to height than those tested by a male experimenter. Although this result was not predicted, we are inclined to believe that such a finding might be related to the well-known effects of experimenter’s sex on participants’ reports of sex-related attitudes and behaviors (e.g., [49]–[52]). For example, just as female researchers elicit more non-traditional responses compared to male researchers [50], the former could also foster the expression of preferences more focused on physical (good genes) rather than psychological (good parent) traits compared to the latter (see [45] for a detailed comparison of good genes and good parent cues). As already proposed for differences related to participants’ sex, we suggest that the scenario manipulation could have masked the probably weaker effects of experimenter’s sex.

Although it would have also been interesting to test whether any of the other scenarios were realistic, it would be extremely challenging to design an experiment to realize a similar task. We cautiously suggest that the findings obtained with the status quo scenario might be generalized to the other scenarios. On the other hand, the fact that participants’ preferences did not differ when comparing two questions phrased using slightly different words indicates that our results were not affected by minor differences (in particular, the presence or absence of a time reference) in the way the question was posed.

## Conclusion

To conclude, our results demonstrate the potential impact of environmental factors – which significantly influence the kinds of trait that people seek in a prospective partner – in determining mate choice. The present research is in line with correlational studies suggesting that situational variables shape mate preferences in a congruous manner [33], [34] (see also [41]). However, unlike correlational studies, our experimental manipulation of virtual scenarios speaks for a direct causal relationship from definite environmental factors to specific mate preferences, corroborating the notion of an evolved plasticity to ecological factors. The specific trade-offs between the various traits according to the different scenarios – in particular, the relative importance attributed by women to dominance in the violence scenario and by men to wealth in the poverty scenario – further corroborate the idea that mate preferences are strongly flexible and affected by contingent needs.

Of course, we cannot – and we do not aim to – exclude the role of genetic factors shaped by environment-specific selection pressures, but our data endorse the idea that the expression of genes affecting reproductive strategies is conditional on the local environment (e.g., see [63]). Furthermore, we do not rule out the potentially crucial influence of the early local environment in shaping adult reproductive strategies (and likely mate preferences), mainly by means of attachment styles [39], [64], [65]. However, although attachment patterns seem to be relatively stable from infancy to adulthood (see [66] for a meta-analysis), our results clearly show that the calibration of reproductive strategies to the local environment is not limited to early developmental phases but also occurs in adulthood (see [67] for consistent considerations), as indirectly suggested by correlational studies.

It is undoubtedly important to bear in mind that our results represent merely ideal preferences in different virtual environments, and cannot fully apply to real life, where ecological context also likely influences a person’s characteristics (e.g., personality and/or perceived mate value). Given that we overtly manipulated environmental conditions only, it could have been difficult for our participants to fully imagine the many (and likely) changes which might occur in themselves – and thus in their mate preferences – under different scenarios. On the other hand, there are two arguments which lead us to think it plausible that our participants’ responses were a reasonable reflection of their likely behavior in the described situations. First, real and hypothetical choices seem to be largely overlapping [68]–[71] and to recruit substantially similar brain areas [72]. Second, the results of the control experiment seem to suggest that our scenarios can be plausibly taken as realistic descriptions of the intended ecological conditions.

## Supporting Information

Appendix S1 **Narratives describing the four scenarios.**
(DOCX)
