# Supplementary material for: Environmental Influences on Mate Preferences as Assessed by a Scenario Manipulation Experiment
Source: PLoS One. 2013 Sep 12;8(9):e74282. doi: 10.1371/journal.pone.0074282 (PMC3771886; doi:10.1371/journal.pone.0074282)
Supplement: Appendix S1 — Narratives describing the four scenarios. (DOCX) [file pone.0074282.s001.docx]

**Appendix S1**

**Status quo (nowadays) scenario:** *“Imagine suddenly finding yourself in a future in which Earth is going through a period of relative sociopolitical stability. State-of-the-art technologies have enabled development of drugs to beat most diseases, but for some of them (among which are several types of tumors) a treatment does not yet exist. The economy is based on industry, agriculture and tourism. However, energy resources are close to exhaustion, and alternative sources are sought. In most countries, a democratic system is in force where politicians are elected by citizens.*

*Although a condition of widespread peace prevails, wars of limited extent still occur among the poorest countries.*

*In such a situation, which characteristics would you prefer in a potential partner?”*

**Violence (post-nuclear) scenario:** *“Imagine suddenly finding yourself in a future in which Earth has been laid waste by a series of nuclear explosions due to a war involving all mankind. The developed world as we know it today no longer exists, and vehicles and fuel are now rarities. Sources of energy are almost vanished, as well as technology and the mass media. The economy is based almost exclusively on agriculture and bartering.*

*There is no longer any form of government or police. As a consequence, many armed gangs go around seeking human settlements in order to pillage the few available resources.*

*In such a situation, which characteristics would you prefer in a potential partner?”*

**Poverty (resource exhaustion) scenario:** *“Imagine suddenly finding yourself in a future in which Earth is going through a long and difficult period of crisis due to the exhaustion of most sources of energy. The industrialized society is almost fully vanished, and the majority of the population is employed in agriculture. Only a minority of very rich individuals have the opportunity to use the few energetic resources left and the technological tools that are so widespread nowadays.*

*As a consequence, there are enormous economic and social differences between the working class and the very few individuals holding power.*

*In such a situation, which characteristics would you prefer in a potential partner?”*

**Prosperity (well-being) scenario:** *“Imagine suddenly finding yourself in a future in which Earth is going through a period of prosperity and well-being. Technology has reached exceptional development, and machines have entirely replaced manual labor. New renewable and non-polluting sources of energy have been found, and transport is extremely safe and fast. Social and economic differences are almost completely vanished, and all individuals enjoy relative prosperity.*

*This has led to a condition of global peace, with the consequent disappearance of economic, social, racial, political and religious conflicts.*

*In such a situation, which characteristics would you prefer in a potential partner?”*
